# Association between Weight Misperception Patterns and Depressive Symptoms in Korean Young Adolescents: National Cross-Sectional Study

**DOI:** 10.1371/journal.pone.0131322

**Published:** 2015-08-27

**Authors:** Haewon Byeon

**Affiliations:** Department of Speech Language Pathology & Audiology, School of Public Health, Nambu University, Gwangju, Republic of Korea; Research and Development Corporation, UNITED STATES

## Abstract

**Purpose:**

Although distortion of weight perceptions has been known as a risk factor for adolescent depression, little has been known about the relationship between weight misperception patterns and depressive symptoms. This study explored the relationship between distortion of weight misperception patterns and depressive symptoms in Korean adolescents.

**Methods:**

The subjects of this study were 109,373 middle school students who participated in the Korea Youth Risk Behavior Web-based Survey (KYRBWS) from 2011 to 2013. By comparing the difference between Body Mass Index (BMI) and subjective perceptions of body weight, misperceptions of weight were classified into two categories: misperception of underweight and misperception of overweight.

**Results:**

When confounding variables were adjusted, the results of the logistic regression analysis revealed that male students who perceived themselves as underweight despite their normal weight were 110% more likely to have depressive symptoms (OR = 1.10, 95% CI: 1.03–1.18) than male students with accurate weight perceptions. On the contrary, for female students, misperceptions of underweight had no significant relationship with depression symptoms. Regarding misperceptions of overweight, female students who perceived themselves as overweight despite their normal weight were 107% (OR = 1.07, 95% CI: 1.02–1.11) more likely to have depressive symptoms than female students with accurate weight perceptions. Moreover, female students who perceived themselves as overweight when they were underweight were 137% (OR = 1.37, 95% CI: 1.18–1.58) more likely to have depressive symptoms.

**Conclusion:**

Male students who underestimate their body weight and female students who overestimate their body weight were at a greater risk of depression emotions than students with accurate weight perceptions.

## Introduction

Over the last two to three decades, adolescent obesity has increased all over the world. In the US, as of 2010, the prevalence rate of adolescent (age 12–19) obesity was 18%, over three times that of 1980. For the same period, in Spain, the UK, and Italy, the prevalence rate of adolescent obesity increased 3 times [1, 2]. Adolescent obesity has been rapidly increasing in Korea as well. As of 2012, the prevalence rate of adolescent (age 12–18) obesity was 14.1% in Korea, 1.5 times that of 2001 (9.4%). In particular, the prevalence rate of obesity in high school students was 18%, the highest among OECD countries [3]. The social and economic cost of child and adolescent obesity was estimated to be USD $13 billion as of 2007 or 5% of total costs for diseases, and among all health risk factors, obesity is the third most common only after smoking and drinking [4].

Adolescent obesity is linked to not only diabetes and stroke in the later years [5, 6] but also psychiatric disorders, such as major depressive disorders [7, 8]. In extreme cases, severe obesity may cause depression symptoms and even suicidal ideation in adolescents [9]. In particular, when puberty begins, young teens (12–14 years of age) undergo radical physical and mental change and show more concern about their body image. Thus, obesity is an important issue for young teens in emotional terms [10]. Although many studies have identified a positive relationship between obesity and depression symptoms [11, 12], the relationship is still unclear. There have been reports that there is no relationship between obesity and depression [13], and a recent systematic review [14] concluded that there are no clear grounds to corroborate a causal relationship between obesity and depression.

Meanwhile, more recent studies have reported that in terms of psychological health, subjects’ perceptions of their body type have more to do with depression than actual body type based on the Body Mass Index (BMI) [15–17]. According to these studies, in today’s appearance-oriented society, adolescents’ negative emotions toward their own bodies lower their self-esteem and thereby cause depressed emotions rather than obesity itself. Especially, when an adolescent's perception of being overweight continues for a number of years, it is highly likely to lead to the risk of depression [16]. In addition, as depression in adolescence has high possibility of developing into a chronic one and continuing into adulthood [18], understanding the relationship between subjective perception of body types and depression symptoms is very important in terms of mental health of adolescents.

Previous studies, however, have examined the relationship between weight misperceptions and depression by merely dichotomizing the misperceptions (accurate vs. inaccurate, overestimated vs. underestimated) [19, 20], and no study has tried investigating the relationship between various patterns of weight misperceptions and depression. For example, even within a group that overestimates their weight, the relationship with depression might differ depending on whether the individuals are actually underweight or normal weight. If the relationship between the various patterns of weight misperceptions and depression can be elucidated through a large-scale epidemiological study, a more effective health policy can be provided.

This study investigated the relationship between body weight misperception patterns and depressive symptoms using nationally representative data collected from Korean adolescents.

## Methods

### Study Population

The data for this study were obtained from the Korea Youth Risk Behavior Web-based Survey (KYRBWS) 2011–2013, a nationwide survey on the health behaviors of Korean adolescents aged 12–18 years conducted jointly by the Korea Centers for Disease Control and Prevention; the Ministry of Health and Welfare; and the Ministry of Education, Science, and Technology [21]. The KYRBWS was approved by the Institutional Review Board of the Korea Centers for Disease Control and Prevention (approval no. 2013-05CON-08). The survey samples included 116,861 students from 400 middle schools selected through stratification, allocation, and extraction sampling. A population was stratified by 43 regional and school-type variables, and then the sample was selected through two stages of school and class. The survey procedures were designed to protect student privacy by allowing anonymous and voluntary participation. Participants were given identification numbers and guaranteed anonymity. After the survey had been fully explained and all participants had provided written informed consent (both directly and from their parents or legal guardians), participants completed an online, self-report questionnaire in a school computer room.

Of these, 112,301 (96.1%) completed this study. Of these, 2,928 students who did not provide weight and height information were excluded, leaving a total of 109,373 students (56,252 boys, 53,121 girls) in the final analysis.

### Measurement

#### Actual body type

The height and weight of the subjects were examined. The BMI, calculated as the weight in kilograms divided by the square of height in meters. Actual body type based on BMI, by applying the Asia-Pacific standard of the WHO suitable for Koreans, was classified into underweight (< 18.5), normal (18.5–24.9), and overweight (> 25).

#### Weight misperception patterns

This study defined the different cases between actual body type on BMI and subjective perception as ‘weight misperception’. Subjective perceptions of body type were surveyed with a question (“What body type do you think you have?”) using a 5-point Likert-type scale from “very thin” to “very fat” and reclassified into “underweight (very thin, thin),” “normal,” and “overweight (fat, very fat).” Patterns of weight misperception were classified based on following procedure. First, weight misperceptions were classified into misperceptions of underweight and misperceptions of overweight by comparing actual body types with subjective perceptions. Subjects who correctly estimated their body weight status were classified as students with “accurate perceptions.” Next, to analyze the relationship between the various patterns of weight misperceptions and depression symptoms, misperceptions of underweight were classified into several categories. These included “perception of self as underweight by those with normal weight,” “perception of self as normal weight by overweight individuals,” and “perception of self as underweight by overweight individuals.” Conversely, misperceptions of overweight were classified into “perception of self as overweight by those with normal weight,” “perception of self as normal weight by underweight individuals,” and “perception of self as overweight by underweight individuals.”

#### Experience of depressive symptoms

According to the standard of ICD–10–DCR, experience of depressive symptoms was defined as sustenance of more than 2 out of 3 symptoms of depressed mood, loss of interest and fatigability for at least 2 weeks over the last 12 months.

#### Covariables

The covariables included in the model were sex, school year, socioeconomic level (high, medium, low), scale of area of residence (large city, small city, countryside), academic achievement (high, medium, low), self-reported happiness (happy, medium, unhappy), self-reported health status (good, normal, poor), smoking, and alcohol consumption.

### Statistical Analysis

Chi-square tests were used to compare characteristics of subjects based on depressive symptoms. The relationship between weight misperceptions and depressive symptoms was represented by an odds ratio and 95% confidence interval using hierarchical logistic regression analysis. Regression models were composed of a first model adjusted only by sociodemographic factors (gender, grade, school type, socioeconomic level, scale of area of residence), a second model adjusted by academic achievement in addition to sociodemographic factors, and a third model adjusted by all confounding factors including health variables (subjective health status, subjective level of happiness, smoking, drinking). IBM SPSS 21.0 (IBM, Inc., Chicago, Illinois) was used for all analyses.

## Results

### General Characteristics of Subject based on Depressive Symptoms

The general characteristics of subjects based on depressive symptoms are presented in Table 1. Results of the Chi-square analysis revealed that adolescents showed significant differences depending on their sex, grade, household’s economic level, scale of area of residence, academic achievement, subjective health status, subjective level of happiness, smoking, drinking, BMI, and weight perceptions. Girls (34.9%); third-grade middle school students (31.0%); students with a low socioeconomic level (38.8%), low academic achievement (35.6%), self-reported unhappiness (63.2%), self-reported poor health (51.1%), and self-reported overweight (31.6%); those currently smoking (40.9%); and those currently drinking (38.2%) showed higher rates of depressive symptoms (p<0.05).

**Table 1:** General characteristics of subjects based on depressive symptoms, n (%).

Variables	Depressive symptoms*		p
	No (n = 77,676)	Yes (n = 31,697)	
Sex			<0.001
Male	43,103 (76.6)	13,149 (23.4)	
Female	34,573 (65.1)	18,548 (34.9)	
School year			<0.001
7	26,789 (73.6)	9,628 (26.4)	
8	25,640 (70.5)	10,747 (29.5)	
9	25,247 (69.0)	11,322 (31.0)	
Socioeconomic level			<0.001
High	29,220 (74.3)	10,118 (25.7)	
Median	37,060 (72.1)	14,365 (27.9)	
Low	11,396 (61.2)	7,214 (38.8)	
Scale of area of residence			0.001
Large city	34,709 (70.6)	14,460 (29.4)	
Small city	33,103 (71.6)	13,129 (28.4)	
Countryside	9,864 (70.6)	4,108 (29.4)	
School record			<0.001
High	31,294 (76.0)	9,863 (24.0)	
Median	20,449 (73.2)	7,503 (26.8)	
Low	25,933 (64.4)	14,331 (35.6)	
Self-reported happiness			<0.001
Happy	53,079 (80.5)	12,862 (19.5)	
Medium	19,908 (64.9)	10,776 (35.1)	
Unhappy	4,689 (36.8)	8,059 (63.2)	
Self-reported health status			<0.001
Good	58,178 (74.9)	19,454 (25.1)	
Normal	16,718 (64.2)	9,336 (35.8)	
Poor	2,780 (48.9)	2,907 (51.1)	
Smoking			<0.001
No	66,351 (73.5)	23,864 (26.5)	
Yes	11,325 (59.1)	7,833 (40.9)	
Alcohol consumption			<0.001
No	55,054 (75.6)	17,737 (24.4)	
Yes	22,622 (61.8)	13,960 (38.2)	
BMI			<0.001
Underweight	26,463 (71.9)	10,344 (28.1)	
Normal	45,186 (70.4)	19,001 (29.6)	
Overweight	6,027 (71.9)	2,352 (28.1)	
Self-reported weight			<0.001
Underweight	22,806 (72.1)	8,823 (27.9)	
Normal	27,833 (72.8)	10,389 (27.2)	
Overweight	27,037 (68.4)	12,485 (31.6)	

* Experience of daily sadness and despair to the degree of interrupting one’s daily routine that has lasted for the last 2 weeks to 12 months.

### Relationship among BMI, Weight perceptions, and Depressive Symptoms

The relationship between body weight based on BMI and depression symptoms is presented in Table 2. Results of the adjustment with sociodemographic variables and academic performance (Model 2) revealed that underweight adolescents were less likely to have depressive symptoms than adolescents with normal BMI (OR = 0.97, 95% CI: 0.94–0.99). However, results of the adjustment with all confounding variables including subjective perception of weight showed that body type based on BMI was not significantly related with depression symptoms.

**Table 2:** The relationship among BMI, subjective perception of weight, and depression symptoms, odds ratio and 95% CI.

Variables	Model 1	Model 2	Model 3
	OR (95% CI)	OR (95% CI)	OR (95% CI)
BMI			
Normal	1	1	1
Underweight	0.97* (0.94, 0.99)	0.97* (0.94, 0.99)	1.01 (0.97, 1.03)
Overweight	1.03 (0.98, 1.09)	0.99 (0.95, 1.05)	0.98 (0.92, 1.03)
Weight perception			
Normal	1	1	1
Underweight	1.12* (1.08, 1.15)	1.12* (1.08, 1.16)	1.10* (1.06, 1.14)
Overweight	1.21* (1.17, 1.24)	1.19* (1.15, 1.23)	1.09* (1.05, 1.12)

*p<0.05

Model 1: Adjusted for sex, age, socioeconomic level, and scale of area of residence.

Model 2: Additionally adjusted for academic achievement.

Model 3: Additionally adjusted for self-reported happiness, self-reported health status, smoking, and alcohol consumption.

The relationship between subjective perceptions of weight and depression symptoms is presented in Table 2. When all confounding variables were adjusted (Model 3), adolescents with misperceptions of underweight were 110% more likely to exhibit depression symptoms (OR = 1.10, 95% CI: 1.06–1.14) than adolescents who perceived themselves as normal weight, while adolescents with misperceptions of overweight were 109% more likely to exhibit depression symptoms (OR = 1.09, 95% CI: 1.05–1.12).

### Relationship between Weight Misperception Patterns and Depression Symptoms

When all confounding variables were adjusted, misperception of underweight was found to be significantly related with depression symptoms (Table 3). The results of a stratified analysis on gender showed that male adolescents who perceived themselves as underweight even when their weight was normal were 110% (OR = 1.10, 95% CI: 1.03–1.18) more likely to exhibit depression symptoms than male adolescents with accurate weight perceptions. On the other hand, for female adolescents, there was no significant relationship between subjective perceptions of underweight and depression symptoms.

**Table 3:** The relationship between distortion of weight misperception patterns and depression symptoms, odds ratio and 95% CI.

BMI	Weight perception	Adjusted model		
		Total	Boys	Girls
		OR (95% CI)	OR (95% CI)	OR (95% CI)
Underestimate				
Normal	Underweight	1.12* (1.05, 1.18)	1.10* (1.03, 1.18)	1.09 (0.97, 1.23)
Overweight	Normal	0.96 (0.68, 1.35)	1.01 (0.67, 1.47)	0.75 (0.33, 1.74)
Overweight	Underweight	1.85 (0.84, 4.08)	1.58 (0.68, 3.71)	6.23 (0.52, 74.91)
Overestimate				
Normal	Overweight	1.05* (1.02, 1.08)	1.03 (0.98, 1.08)	1.07* (1.02, 1.11)
Underweight	Normal	0.95 (0.91, 1.01)	0.79* (0.73, 0.87)	1.05 (0.98, 1.12)
Underweight	Overweight	1.25* (1.11, 1.41)	1.04 (0.84, 1.30)	1.37* (1.18, 1.58)

Reference group is accurate weight perception

*p<0.05

Adjusted model: Adjusted for school year, socioeconomic level, scale of area of residence, academic achievement, self-reported happiness, self-reported health status, smoking, and alcohol consumption.

Overestimates of weight produced different results depending on gender. Female adolescents who perceived themselves as overweight even when their weight was normal were 107% more likely to exhibit depression symptoms (OR = 1.07, 95% CI: 1.02–1.11) than female adolescents with accurate weight perceptions. Female adolescents who perceived themselves as overweight even when they were underweight were 137% more likely to exhibit depression symptoms (OR = 1.37, 95% CI: 1.18–1.58). On the contrary, male adolescents who perceived themselves as having normal weight even when they were underweight were 21% less likely to exhibit depression symptoms (OR = 0.79, 95% CI: 0.73–0.87) than male adolescents with accurate weight perceptions.

## Discussion

Subjective perceptions of body type were found to be significantly related with depression symptoms in this study. Studies on the relationship between adolescent obesity and depression have mainly used BMI and produced different results. For example, some studies have reported that obesity is related with depression [11, 12], while others have reported that only severe obesity is related with depression [14], and still others have reported that obesity is never related with depression [8]. There are two possible explanations for these discrepancies: confounding variables and a mediating factor such as depression.

First, the variable findings might be due to the effect of confounding variables. Atlantis and Ball [22] pointed out that numerous preceding studies had not adjusted such confounding variables as sociodemographic factors (e.g., gender, socioeconomic level, lifestyle), and they suggested the necessity of studies that consider various factors. In this study, obesity based on BMI was found to be related with depression symptoms when considering only sociodemographic variables, but when all health conditions and health behaviors were considered, the significance of the relationship was lost. Likewise, in the study of Hayes and Ross [23], although obesity and depression were related independently, they were not when all sociodemographic factors and health conditions were considered, as in this study.

Another possible reason for the lack of a significant relationship between obesity and depression is that negative perceptions of and dissatisfaction with one’s own body type caused by obesity may lead to depression rather than obesity itself directly causing depression. A negative body image formed in adolescence when self-identity is actively developed has been shown to be related with mental disorders, such as anxiety and depression [23, 24]. Therefore, rather than weight itself, the dissatisfaction with one’s weight is deemed to negatively affect self-esteem, ultimately leading adolescents to suffer from depressive emotions.

In this study, the relationship between patterns of weight misperceptions and depression symptoms differed depending on gender. In the case of female adolescents, perceptions of overweight were significantly related with depression symptoms regardless of actual weight. Likewise, a study on Chinese adolescents confirmed that perceptions of overweight in females were related with depression [25, 26]. In addition, in the study of Lee et al. [27], which analyzed the relationship between obesity and depression by classifying obesity into BMI and perception of weight, BMI was not significantly related with depression symptoms, and women who perceived themselves as obese when they actually were not had the highest depression scores. Based on these studies, researchers concluded that perceptions of body weight are more important in influencing the depression of adolescents than actual obesity based on body weight. Distortion of weight perception is closely related with depression, because subjective perceptions of body weight are independent of actual obesity [28]. As body image is mostly determined by social standards and women are more sensitive, how one perceives and evaluates one’s body can especially affect depressive feelings [29]. Generally, obese women tend to have a sense of inferiority in terms of their appearances, as they feel they can never achieve the distorted body image preferred by society [29]. Middle school students going through puberty are particularly sensitive to their body weight as evidenced by the result of this study that female students who perceived themselves as obese when they were in fact underweight had a high risk of depression symptoms.

In the case of male students, those who perceive themselves as underweight even when they are of a normal weight are likely to exhibit depression symptoms. The problem of underweight has received comparatively less attention than obesity, and little is known about its emotional aspects [22]. One possible explanation is that a subjective sense of underweight leads to the development of a negative body image just as a subjective sense of overweight. According to a study on body image based on gender, obesity was found to have a negative effect on body image in women, whereas underweight was found to cause dissatisfaction in men [26, 30]. In this study, male students who perceived their body weight as normal when they were in fact underweight were 21% less likely to exhibit depression symptoms than those who had accurate weight perceptions, which shows the dissatisfaction of males with low body weights and awareness of their body type. Therefore, it is possible that male students will experience depression if they perceive themselves as underweight despite their normal weight, as they feel a sense of inferiority due to their body image.

The limitations of this study are as follows: First, depressive symptoms defined in the study were based on the subjects’ reports. Due to the restrictions of the large-scale online survey method, diagnoses of depression by a psychiatrist were not conducted. In order to elucidate the relationship between weight perception patterns and depression symptoms, medical diagnoses using depression test tools are required in the future. Second, there are possibly other confounding variables that can affect depression symptoms in addition to the ones included in this study. In particular, no survey was conducted on the treatment of participants’ depression symptoms; thus, caution is required in the interpretation of the results. Third, the results of this study should not be interpreted as indicating a causal relationship. Longitudinal studies are required to demonstrate a causal relationship in the future.

## Conclusion

Male students who underestimate their body weight and female students who overestimate their body weight are at a higher risk of depressive emotions. Counseling and education are required to encourage adolescents to develop accurate perceptions of their body types and thus protect their mental health.
